# Trends in Incidence and Survival of 1496 Patients with Mucosal Melanoma in The Netherlands (1990–2019)

**DOI:** 10.3390/cancers15051541

**Published:** 2023-02-28

**Authors:** Florine L. Boer, Vincent K. Y. Ho, Marieke W. J. Louwman, Anne M. R. Schrader, Charlotte L. Zuur, Christian U. Blank, Mariette I. E. van Poelgeest, Ellen H. W. Kapiteijn

**Affiliations:** 1 Department of Gynaecology and Obstetrics, Leiden University Medical Centre, 2333 ZA Leiden, The Netherlands; 2 Department of Medical Oncology, Leiden University Medical Centre, 2333 ZA Leiden, The Netherlands; 3 Department of Research and Development, Netherlands Comprehensive Cancer Organisation (IKNL), 3511 DT Utrecht, The Netherlands; 4 Department of Pathology, Leiden University Medical Centre, 2333 ZA Leiden, The Netherlands; 5 Department of Head and Neck Surgery and Oncology, The Netherlands Cancer Institute, 1066 CX Amsterdam, The Netherlands; 6 Department of Otorhinolaryngology, Leiden University Medical Centre, 2333 ZA Leiden, The Netherlands; 7 Department of Medical Oncology, The Netherlands Cancer Institute, 1066 CX Amsterdam, The Netherlands

**Keywords:** mucosal melanoma, incidence, survival, immune and targeted therapy

## Abstract

**Simple Summary:**

Mucosal melanoma (MM) is rare and entails a poor prognosis. MM is biologically different from cutaneous melanoma (CM). For advanced CM, overall survival has improved since the introduction of immune and targeted therapy. In contrast, little is known about the effect of their introduction on the survival of MM. This study presents the incidence, clinical characteristics, treatment characteristics, and survival of MM over 30 years (1990–2019) in the Netherlands. We conclude that the incidence of MM remained stable, and survival has slightly improved when comparing the timeframe 2014–2019 with previous years. However, the prognosis of MM remains poor as compared to CM. Future studies addressing the effect of immune and targeted therapy in MM are needed to improve outcomes for patients with MM.

**Abstract:**

Background: Mucosal melanoma (MM) is a rare tumour with a poor prognosis. Over the years, immune and targeted therapy have become available and have improved overall survival (OS) for patients with advanced cutaneous melanoma (CM). This study aimed to assess trends in the incidence and survival of MM in the Netherlands against the background of new effective treatments that became available for advanced melanoma. Methods: We obtained information on patients diagnosed with MM during 1990–2019 from the Netherlands Cancer Registry. The age-standardized incidence rate and estimated annual percentage change (EAPC) were calculated over the total study period. OS was calculated using the Kaplan–Meier method. Independent predictors for OS were assessed by applying multivariable Cox proportional hazards regression models. Results: In total, 1496 patients were diagnosed with MM during 1990–2019, mostly in the female genital tract (43%) and the head and neck region (34%). The majority presented with local or locally advanced disease (66%). The incidence remained stable over time (EAPC 3.0%, *p* = 0.4). The 5-year OS was 24% (95%CI: 21.6–26.0%) with a median OS of 1.7 years (95%CI: 1.6–1.8). Age ≥ 70 years at diagnosis, higher stage at diagnosis, and respiratory tract location were independent predictors for worse OS. Diagnosis in the period 2014–2019, MM located in the female genital tract, and treatment with immune or targeted therapy were independent predictors for better OS. Conclusion: Since the introduction of immune and targeted therapies, OS has improved for patients with MM. However, the prognosis of MM patients is still lower compared to CM, and the median OS of patients treated with immune and targeted therapies remains fairly short. Further studies are needed to improve outcomes for patients with MM.

## 1. Introduction

Mucosal melanomas (MM) are malignant tumours arising from melanocytes located in the mucosal lining of the head and neck region or the respiratory, gastrointestinal, anorectal, or genital tract [1]. MM is rare and accounts for approximately 1.4% of all melanomas in the Caucasian population. Incidence is higher in the Asian population (23% of all melanomas), boosting research on this entity in this region [2]. MM has a higher incidence in women than men (2.8 cases per million versus 1.8 cases per million). This is partly explained by the mucosal lining in the female genital tract, which comprises 15–20% of all MM [3,4,5,6,7]. Due to its rarity, MM is still poorly understood, and clinical management is mostly based on guidelines for cutaneous melanoma (CM) [8,9,10].

MM has a significantly lower 5-year overall survival (OS) compared to CM (37% versus 92%) [11]. Furthermore, MM entails a lower median OS after the detection of distant spread disease (9.1 versus 11.7 months) [12]. The poor prognosis of MM is assumed to be caused by aggressive tumour behaviour, higher tumour stage at diagnosis, and an often-challenging location for surgical excision, more often leading to incomplete resections. Additionally, MM has a lower tumour mutational burden and may be less immunogenic, which makes the metastatic disease less sensitive to immunotherapy. Compared to CM, MM harbour a BRAF mutation less often (40–50% in CM versus 10% in MM). However, MM more often contain a targetable KIT mutation (2–10% in CM versus 15–39% in MM), although response duration on KIT inhibitors is short [13]. More importantly, a lower PD-1 expression rate (17–29% in MM versus 34% in CM) may affect the potential benefit of immunotherapy [14].

Since its introduction in 2011, immunotherapy with CTLA-4 and PD-1 inhibitors and targeted therapy (BRAF and MEK inhibitors) have completely changed treatment strategies for stage III and IV CM. The effect of these therapies is reflected by an increase in 5-year OS between 2013 and 2016, from 81% to 92% in men and from 88% to 96% in women. This is predominantly due to improved OS in stage II, III, and IV disease [15,16]. Furthermore, as neoadjuvant therapy in both high-risk resectable and locally advanced CM, immunotherapy can result in shrinkage of the primary tumour, facilitating R0 resections and improving surgical morbidity [17].

In contrast to CM, the efficacy of immune and targeted therapy in MM remains unknown, as patients with MM are often excluded from clinical trials. Moreover, it is hypothesised that MM does not benefit from the introduction of immune and targeted therapy as much as CM. This is demonstrated by a recent observational study reporting that the median OS of stage III and stage IV MM did not improve in the time period 2015–2017 compared to 2013–2014 (8.7 months vs. 8.9 months, respectively) [16].

This population-based study reports on long-term trends in the incidence and survival of MM in the Netherlands. We aimed to evaluate whether survival has improved since the introduction of immune and targeted therapies. We estimated the impact of these therapies by assessing the effectiveness of their time of introduction as a proxy for the prognosis of patients with MM. Furthermore, by analysing all stages and all tumour sites of this disease, alternative explanations for the poorer survival of MM compared to CM may be explored.

## 2. Materials and Methods

### 2.1. Patient Selection

We retrieved patient records from the Netherlands Cancer Registry (NCR), hosted by the Netherlands Comprehensive Cancer Organization (IKNL). The NCR is a nationwide population-based registry containing information on patient and tumour characteristics, primary treatment, and survival of all newly diagnosed cases of cancer in the Netherlands since 1989. Follow-up information on the vital status of every patient is obtained through a yearly linkage with the Municipal Personal Records Database (Gemeentelijke Basisadministratie, GBA), with the latest update obtained on 31 January 2022. Primary treatment is registered for therapies provided as part of the initial treatment plan; no information was available on second or higher-line treatment. The study design, data abstraction process, and storage protocols were approved by the national supervisory committee of the NCR.

**Figure 1:**
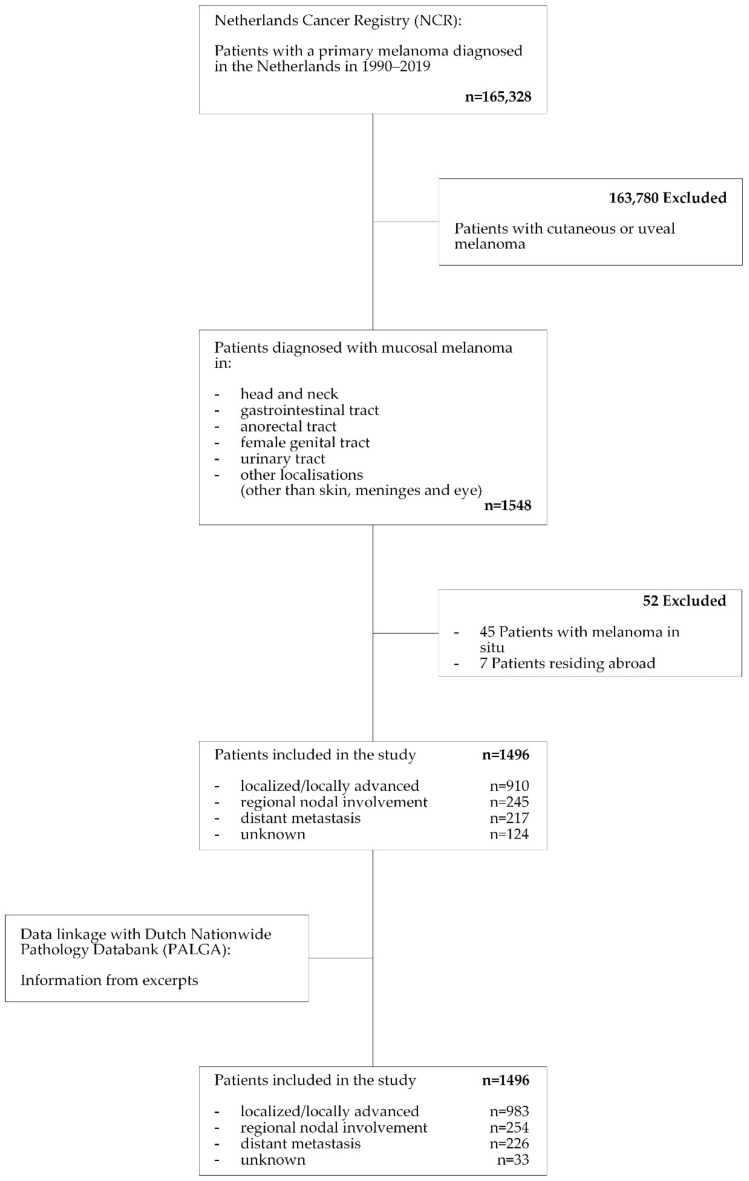
STROBE diagram for case selection for the study (STROBE: Strengthening the reporting of observational studies in epidemiology).

From the NCR database, all patients with a primary MM diagnosed during the period 1990–2019 were selected. Cases were identified based on topography and histology codes of the International Classification of Diseases for Oncology (ICD-O). Patients with melanoma in situ were excluded, as were foreign patients, as the date of death was not available for these patients (Figure 1).

Due to the different staging classifications applied to different tumour locations (e.g., TNM and Extent of Disease), and concurrent changes of the TNM staging system over time, MM were reclassified as local or locally advanced disease, locoregional spread disease, or distant spread disease. Local or locally advanced disease was defined as a disease confined to the primary tumour location and close surroundings. Locoregional spread disease entails being either pathologically or radiologically confirmed as spread to any lymph node(s). Distant spread disease is defined as a disease with either pathologically or radiologically confirmed spread to distant skin, visceral organs, or bone. Given the large proportion of cases with an initially unknown stage (*n* = 124; 8.3%, Table 1), the study database was matched with the Dutch Nationwide Pathology Databank (PALGA) (Figure 1). Based on the detailed information from pathology reports, most of the cases with unknown stages could be reclassified (unknown stage *n* = 33; 2.2%, Table 1). With respect to the tumour site, cases were classified based on the ICD-O code in the head and neck, gastrointestinal tract, anorectal tract, female genital tract, and respiratory tract. The head and neck were subcategorized as oral, sinonasal and pharynx/glottis, female genital tract in vulva, vagina, and other, and anorectal in the anus and rectum (Table S1). As immunotherapy and targeted therapy could only be reliably distinguished from one another for the most recent years, they were grouped together for all analyses.

### 2.2. Statistical Analysis

To assess trends over time, cases of MM were analysed according to 6-year time periods based on their year of diagnosis, with an additional focus on comparing the latest period (2014–2019) with all previous years. This cut-off was chosen since, in our population, immune and targeted therapies were introduced in clinical practice from 2014 onwards. We analysed the following variables: sex, age, tumour site, tumour stage, type of hospital at the time of diagnosis (academic centre, general hospital), and primary treatment (surgery, radiotherapy, chemotherapy, and targeted therapy and immunotherapy grouped together).

Normally distributed continuous data were reported as means with standard deviations and skewed distributions as medians with interquartile ranges. Differences between descriptive variables were tested with the Chi-square test, Fisher’s exact test, or the independent t-test.

Reporting on incidence, annual rates per 100,000 person-years with corresponding 95% confidence intervals (95% CI) were calculated using the average annual population provided by Statistics Netherlands (Centraal Bureau voor de Statistiek, CBS). The rates were age-adjusted through standardization to the European standard population (European Standardized Rate, ESR). Trends in incidence were evaluated through the Estimated Annual Percentage Change (EAPC).

OS was calculated using the Kaplan–Meier method. Differences in survival curves between groups were assessed with log-rank tests. Relative survival (RS) was calculated by matching observed OS in patients to expected survival in the general Dutch population summarized in annual life tables on age, gender, and calendar year (retrieved from CBS) using the Pohar-Perme estimator. Independent predictors for OS were evaluated by applying multivariable Cox proportional hazards regression models, following the selection of potential predictors based on a *p*-value of <0.1 in univariable analyses. All statistical analyses were two-sided, with a *p*-value <0.05 being considered significant. Analyses were performed using software packages IBM SPSS Statistics version 20.0 and Stata version 17.0 (StataCorp, College Station, TX, USA).

## 3. Results

### 3.1. Incidence

Between 1990 and 2019, 1496 patients were diagnosed with MM in the Netherlands (Table 1). MM was more prevalent in women than men (73.2% versus 26.8%). The median age at diagnosis was 72 years, with an interquartile range (IQR) of 62–81 years. Most MM were located in the female genital tract (*n* = 640; 42.8%) and the head and neck region (*n* = 505; 33.8%), and the majority concerned local or locally advanced disease (*n* = 983; 65.7%). The majority of cases in the head and neck region (79.8%), the female genital tract (67.7%), and the urinary tract (68.8%) presented as local or locally advanced diseases (Table 2). Anorectal and gastrointestinal diseases were more likely to present at a higher stage at diagnosis, i.e., locoregional spread (28.2% and 13.2%, respectively) or distant spread disease (29.4% and 51.3%, respectively). Over the total study period, the proportion of local or locally advanced diseases decreased from 73.2% in 1990–1995 to 63.5% in 2014–2019. The distribution of stage at diagnosis was not significantly different in 2014–2019 compared to all previous years.

Over three-quarters of all patients underwent surgery (*n* = 1152; 77.0%). Radiotherapy was part of the primary treatment in 30.7% of cases, while systemic therapy was part of the initial treatment in 5.8% (Table 1). Half of the patients who received systemic therapy did not have surgery or radiotherapy (data not shown). The majority of the patients with local or locally advanced disease underwent surgery (86.9%, *n* = 854) or radiotherapy (33.3%, *n* = 326). Systemic treatment was not often part of the initial treatment in this stage (Table S2). Surgery and radiotherapy were also the main treatment strategies in locoregional spread disease (respectively 79.9%, *n* = 203 and 28.7%, *n* = 73). Patients with distant spread disease received various types of treatment, of whom 28.3% were systemic treatments. Of these patients, only 16.8% received immune and/or targeted therapy.

Compared to previous years, patients diagnosed in 2014–2019 underwent surgery less often (70.9% versus 79.1%; *p* < 0.01). This was the case for patients with local or locally advanced disease (82.6% versus 88.3%; *p* = 0.03) and those with distant spread disease (23.1% versus 37.3%; *p* = 0.04), but not for patients with locoregional spread disease (77.1% versus 81.0%; *p* = 0.50) (data not shown). Overall, the first surgery took place in one of the academic centres more often (56.7% versus 50.3%; *p* = 0.04). Systemic therapy was initially provided in 5.8% of patients, but before 2014, this mainly consisted of chemotherapy (34/36 patients). Immune and targeted therapy were more often provided as part of primary treatment in 2014–2019 compared to all years before 2014 (13.4% vs. 0.2% of cases (*p* < 0.01)).

**Figure 2:**
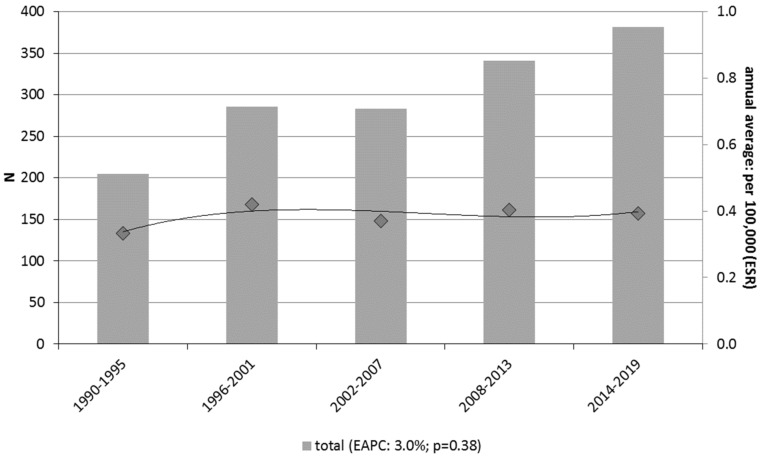
Crude numbers (bars, left axis) and annual averaged, age-adjusted incidence rates (line, right axis) for patients with mucosal melanoma in the Netherlands.

The number of MM patients increased from 205 cases in 1990–1995 to 381 in 2014–2019 (Figure 2). The age-adjusted incidence rate remained stable over time, estimated at 0.33 per 100,000 ESR in 1990–1995 and 0.39 per 100,000 ESR in 2014–2019 (EAPC 3.0%, *p* = 0.38).

### 3.2. Survival

Overall, patients with MM had a 1-, 2-, and 5-year OS of 67.2% (95%CI: 64.7–69.5%), 44.4% (95%CI: 41.9–46.9%), and 23.8% (95%CI: 21.6–26.0%), respectively, with median OS of 1.7 years (95%CI: 1.6–1.8) (Table 3). OS differed across tumour stages, with 5-year OS rates of 30.8% for patients with local or locally advanced disease (95%CI: 27.9–33.7%), 14.0% for patients with locoregional spread disease (95%CI: 10.0–18.8%), and 5.2% for those with distant spread disease (95%CI: 2.8–8.8%). Accordingly, median OS was 2.4 years (95%CI: 2.1–2.7), 1.3 years (95%CI: 1.1–1.6), and 0.6 years (95%CI: 0.4–0.7), respectively. OS was relatively higher for MM of the urinary tract (5-year OS 31.3%, 95%CI: 11.4–53.6%), the head and neck region (24.7%, 95%CI: 20.9–28.6%), and the female genital tract (5-year OS 27.8%, 95%CI: 24.4–31.4%), and within the latter site, prognoses differed significantly for specific subsites. Median OS for patients with MM located at the vulva was 2.9 years (95%CI: 2.5–3.4), while this was 1.1 years (95%CI: 1.0–1.4) for those with MM located in the vagina (Table 3). The 5-year RS for all patients with MM was 29.0% (95%CI: 26.2–31.8%).

**Figure 3:**
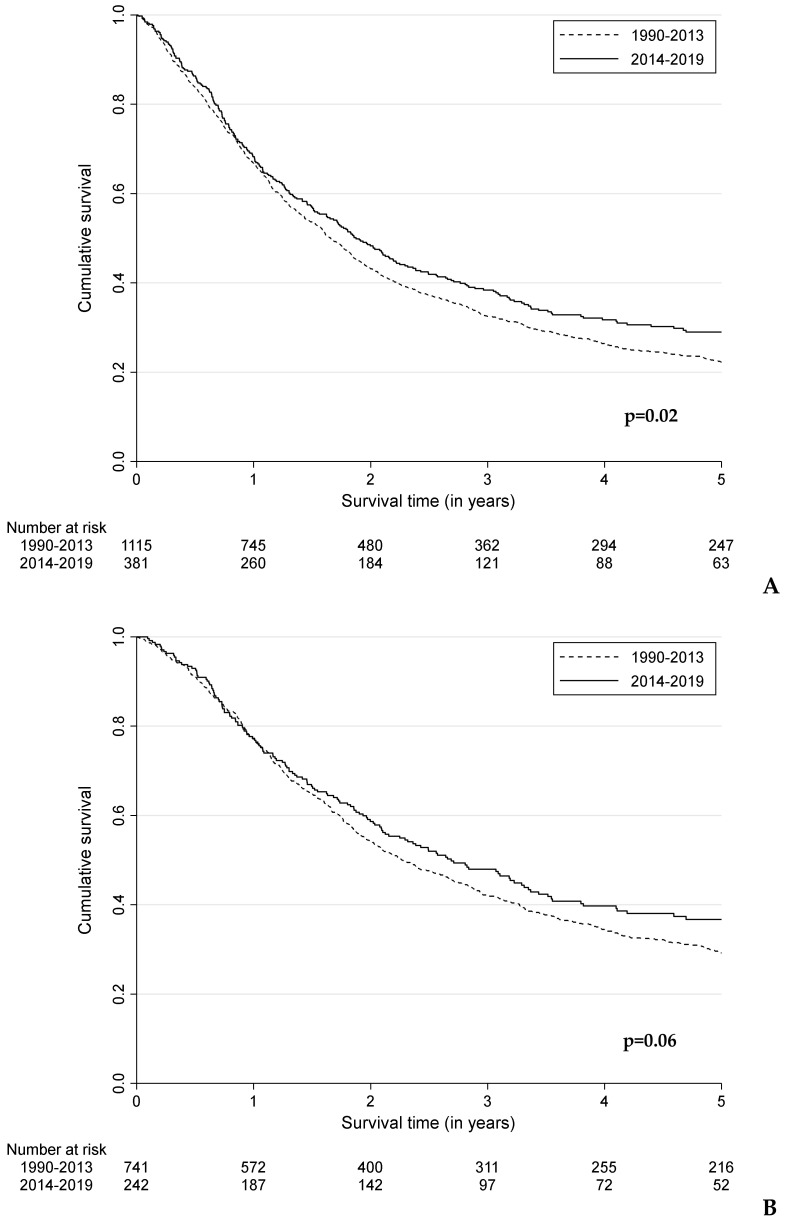

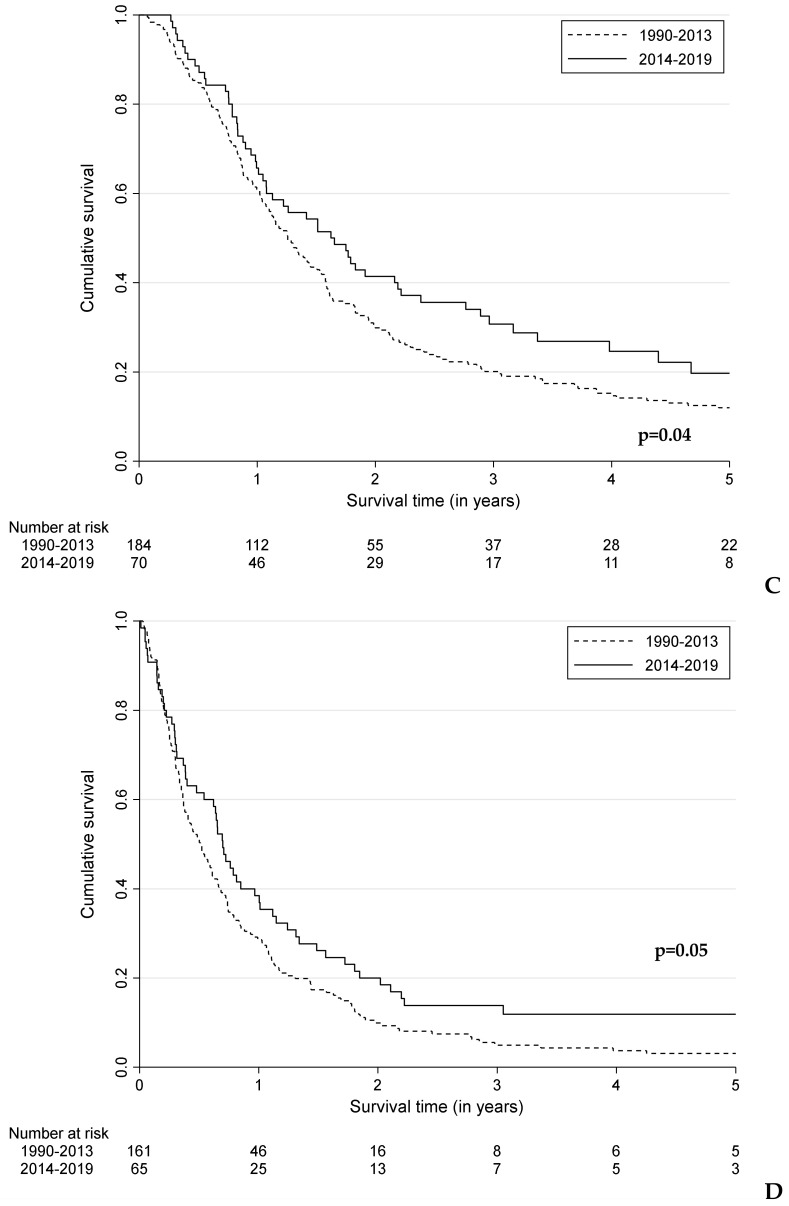
Kaplan–Meier curves representing the overall survival of patients with MM according to the period of diagnosis for (**A**) the total population, (**B**) local or locally advanced disease, (**C**) locoregional spread disease, and (**D**) distant spread disease.

Compared to the period 1990–2013, patients diagnosed in 2014–2019 had a better 5-year OS (*p* = 0.02), without significant improvement in median OS: 1.9 years (95%CI: 1.6–2.2) versus 1.6 years (95%CI: 1.5–1.8), respectively (Figure 3A). At 5 years, OS was 29.0% (95%CI: 24.2–33.9%) compared to 22.3% (95%CI: 19.9–24.8%) for the periods 1990–2013 and 2014–2019 (data not shown). OS improved across all tumour stages, but only significantly for locoregional spread disease (*p* = 0.04) (Figure 3B–D). For these patients, 5-year OS was 19.7% (95%CI: 10.4–31.1%) in 2014–2019 compared to 12.0% (95%CI: 7.8–17.1%) in 1990–2013. For patients with distant spread disease, the 5-year OS was 11.9% (95%CI: 5.3–21.2%) in 2014–2019 compared to 3.1% (95%CI: 1.2–6.7%) in 1990–2013 (Figure 3D).

### 3.3. Predictors for Survival

Univariable analysis showed that diagnosis between 2014–2019, female sex, surgery as primary treatment, and MM located at the female genital tract were associated with better survival (Table 4). Higher age, gastrointestinal, anorectal or respiratory location, higher stage at presentation, radiotherapy, chemotherapy, or immune and targeted therapy as primary treatment were associated with worse survival. Multivariable analysis showed that respiratory location, higher age, and higher stage at presentation were independently associated with worse OS. Diagnosis in the period 2014–2019 was associated with better OS compared to diagnosis between 1990–2013 (Table 4) (HR 0.82 (95%CI: 0.71–0.95; *p* < 0.01). Other factors that were significantly associated with a better prognosis in multivariable analysis were patients’ younger age, MM located in the female genital tract, local or locally advanced disease, and initial provision of immune or targeted therapy. Patients who received immune or targeted therapy had an HR of 0.60 (95%CI: 0.42–0.86; *p* = 0.01) compared to those who were not treated with immune or targeted therapy. Although surgery showed a significant effect in both univariable and multivariable analyses, the proportional hazards assumption was considered violated, and the estimates of the definitive model were stratified for this variable.

## 4. Discussion

This large retrospective population-based study analysing real-world data of stage I-IV MM in the Netherlands from 1990–2019 shows that despite the introduction of immune and targeted therapies, survival of MM remains poor. The 5-year OS is 23.8%, and the indisputable aggressive course of the disease is reflected by the short median survival of 1 year and 8 months. Though survival has improved when comparing timeframes before and after the introduction of immune and targeted therapies, the absolute survival benefit seems fairly limited (1 year and 7 months vs. 1 year and 10 months for all stages). For patients with regional or distant spread disease, improvement was limited to 2 months only.

In our study, the mean age-adjusted incidence rate for MM over the total period was 0.38 per 100.000 person-years and remained stable over time. These findings are in line with a large Survival, Epidemiology, and End Results (SEER) database, which included CM and MM patients between 1973 and 2013 in the United States of America (totaling 133.996 patients, of which 1522 had MM), also showing increasing incidence and improved survival over time for CM whilst incidence for MM remained stable, and survival remained poor [11]. The same trend of increasing incidence and higher survival rates for CM, particularly for stage II, III, and IV disease, was observed in Dutch epidemiologic research with data from 2003 to 2018. The median OS of advanced CM increased from 11.3 to 16.9 months, whilst the median OS of advanced MM did not improve when comparing the same timeframes (2013–2014 vs. 2015–2017) [15,16]. As immune and targeted therapy were introduced in 2011, data on this subject should be read with caution as the absolute number of patients treated with these therapies in studies are low. Our study confirms the unfavourable prognosis of MM compared with CM [12]. However, there is a significant improvement in survival over all stages, and specifically, the locoregional spread of disease, when comparing 2014–2019 with all previous years. Moreover, a trend towards better survival was seen for local or locally advanced disease and the distant spread of disease.

Multivariable analysis showed that diagnosis during the timeframe 2014–2019 is independently associated with better OS. This may be explained by the application of immune and targeted therapy as second or later-line treatment. In this study, we only had access to the stage at initial diagnosis and first-line therapy. However, recurrence rates are high in MM and most often recur as regional or distant spread disease [18,19]. We hypothesise that patients included in this study may have received immune and targeted therapies following disease progression or recurrence. As such, the benefit of these therapies may be expressed directly in our analysis as well as through diagnosis during 2014–2019. In addition to the timeframe 2014–2019, other independent factors associated with better survival were treatment with immune or targeted therapies and MM located in the female genital tract.

Data regarding the location of MM as a predictor of survival are inconsistent. Large studies, including 704 and 1814 MM, demonstrated no difference in survival between MM originating from various locations, even when correcting for the stage of the disease. In contrast, other studies associated MM located in the female genital tract or head and neck with better OS, while the latter was also reported to have worse survival compared to other locations [20,21,22,23,24,25]. We found that MM of the female genital tract and the head and neck more often present with localised disease, corresponding with higher survival rates compared with other locations of MM. Within MM of the female genital tract, the better prognosis of vulvar MM compared to vaginal MM is in line with the literature [26,27]. Vulvar and vaginal MM are often classified as one entity. However, the vulva consists of both cutaneous and glabrous skin, whilst the vagina only consists of glabrous skin with a mucosal lining. MM originating from cutaneous and not mucosal lining and a more visible location allow vulvar MM to be diagnosed at an earlier stage than vaginal MM, which may contribute to a better prognosis. Moreover, a mutational analysis of 95 female genital tract melanomas showed that BRAF mutation, which is often found in CM, is more often detected in vulvar MM compared with vaginal MM, respectively, in 28% and 9% of cases [26]. These data suggest that MM located at the vulva may even have more resemblance to CM than with MM and that immune and targeted therapy may likewise be promising for advanced disease. We suggest that vulvar and vaginal melanomas should not be classified as one entity, given their distinct origin with different prognoses.

Immune and targeted therapy are the cornerstone of advanced CM treatment nowadays since they demonstrate better and more durable response rates and better long-term outcomes than chemotherapy [28]. In the Netherlands, immune and targeted therapy for advanced CM have been available since 2011 and have been used in clinical practice since 2014 [16]. In contrast, data on these therapies in MM is limited, and few patients with MM have been treated with immune and targeted therapies. Additionally, studies are mostly retrospective, and in the case of a prospective set-up, follow-up is short. A retrospective multicentre international study and a multicentre Japanese study including 545 and 329 advanced or unresectable stage II MM treated with anti-PD1 (pembrolizumab) alone or combined with anti-CTLA-4 (ipilimumab) state that these therapies have lower efficacy than in CM (response rate of 30% and 26% in MM), and that response is also less durable (mean duration of response (mDoR) is 25 months) [29,30]. Moreover, the 5-year follow-up of 79 patients with MM treated with anti-PD-1, anti-CTLA-4, or a combination (ipilimumab and/or nivolumab) in Checkmate 067 showed poor long-term efficacy for either of these agents [31]. In contrast with CM, there is no difference in progression-free or overall survival when comparing combined anti-PD-1 and anti-CTLA-4 therapy or anti-PD-1 monotherapy with pembrolizumab. Evidence of anti-PD-1 therapy in advanced CM demonstrates a response rate of 42% and mDoR of 52 months [32,33]. Data on MM treated with anti-CTLA-4, anti-PD-1, or a combination of both agents show a median OS of 9.6, 11.5, and 11 months which is comparable with the 55 patients in our cohort who were treated with either immune or targeted therapy and had median OS of 12 months [16,34]. Whilst we have no definite data on the type of systemic therapy, we are certain that the majority of these patients are treated with immune therapy and not with targeted therapy. This is endorsed by a Dutch paper which published treatment data of advanced MM from 2013–2017, of which 76.4% of the first-line systemic treatment consisted of ipilimumab or nivolumab [16].

Though the role of immunotherapy in MM is still controversial, this could be beneficial in resectable or bulky MM, given the promising results of neoadjuvant immunotherapy in CM. This treatment strategy may contribute to less invasive surgery in anatomically challenging locations and possibly reduce significant morbidity. Only one retrospective study analysed neoadjuvant immunotherapy in MM and demonstrated a pathological response rate of 35% (11/31), of which three patients did not require surgical treatment and had an ongoing response [35]. Further research, including prospective data on this subject, is needed.

The observational set-up of this study warrants some caution in interpreting the results presented here. In addition, as information on recurrences, progression of the disease, and associated treatment was not available, progression-free survival could not be analysed. Unfortunately, as immune and targeted therapy could only be reliably distinguished from one another in more recent years, we were unable to evaluate the independent efficacy of immune and targeted therapy. Despite these limitations, we presume that this study established valuable additions to current knowledge on MM by providing real-world data on incidence and survival in a large cohort over a 30-year time period.

## 5. Conclusions

In conclusion, the incidence of MM has remained stable over the last 30 years, whilst overall survival has slightly improved since the introduction of immune and targeted therapy. However, the median survival remains fairly short, especially as compared to CM, reflecting the poor prognosis of this aggressive cancer type. Future studies examining the effect of immune and targeted therapies in MM are highly needed. Therefore, considering the rarity of MM, we advocate international multicentre collaborations and the inclusion of patients with MM in clinical trials.

## Figures and Tables

**Table 1:** Baseline, tumour, and treatment-related characteristics of patients with mucosal melanoma in the Netherlands.

	Total		1990–2013		2014–2019		
	*N* = 1496		*N* = 1115		*N* = 381		
	*n*	%	*n*	%	*n*	%	*p*
**Sex**							0.43
Male	401	26.8%	293	26.3%	108	28.3%	
Female	1095	73.2%	822	73.7%	273	71.7%	
**Age at diagnosis (years)**							0.32
0–59	323	21.6%	242	21.7%	81	21.3%	
60–69	297	19.9%	209	18.7%	88	23.1%	
70–79	429	28.7%	324	29.1%	105	27.6%	
≥80	447	29.9%	340	30.5%	107	28.1%	
Median (interquartile range)	72 (62–81)		73 (62–81)		71 (62–80)		
**Tumour site**							**0.04**
Head and neck	505	33.8%	380	34.1%	125	32.8%	
Gastrointestinal tract	76	5.1%	51	4.6%	25	6.6%	
Anorectal tract	248	16.6%	176	15.8%	72	18.9%	
Female genital tract	640	42.8%	488	43.8%	152	39.9%	
Urinary tract	16	1.1%	9	0.8%	7	1.8%	
Respiratory tract	11	0.7%	11	1.0%	0	0.0%	
**Tumour stage**							0.15
Local/locally advanced disease	983	65.7%	741	66.5%	242	63.5%	
Locoregional spread disease	254	17.0%	184	16.5%	70	18.4%	
Distant spread disease	226	15.1%	161	14.4%	65	17.1%	
Unknown	33	2.2%	29	2.6%	4	1.0%	
**Surgery**							**<0.01**
No	344	23.0%	233	20.9%	111	29.1%	
Yes	1152	77.0%	882	79.1%	270	70.9%	
**Hospital of first surgery**							0.04 **
Academic centre	504	43.8%	351	39.8%	153	56.7%	
General hospital	459	39.8%	347	39.3%	112	41.5%	
Unknown	189	16.4%	184	20.9%	5	1.9%	
**Radiotherapy**							0.38
No	1036	69.3%	779	69.9%	257	67.5%	
Yes	460	30.7%	336	30.1%	124	32.5%	
Systemic therapy *							**<0.01**
No	1409	94.2%	1079	96.8%	330	86.6%	
Yes	87	5.8%	36	3.2%	51	13.4%	
** Chemotherapy**							**<0.01**
No	1462	97.7%	1081	97.0%	381	100.0%	
Yes	34	2.3%	34	3.0%	0	0.0%	
** Immune and targeted therapy**							**<0.01**
No	1443	96.5%	1113	99.8%	330	86.6%	
Yes	53	3.5%	2	0.2%	51	13.4%	
**Hospital of first contact**							0.43 **
Academic centre	202	13.5%	155	13.9%	47	12.3%	
General hospital	1291	86.3%	957	85.8%	334	87.7%	
Unknown	3	0.2%	3	0.3%	0	0.0%	

* Only primary therapy is listed; ** test academic centres versus general hospitals.

**Table 2:** Distribution of tumour stage by site of mucosal melanoma.

Tumour Site	Total		Local/Locally Advanced Disease		Locoregional Spread Disease		Distant Spread Disease		Unknown	
	1496		65.7%		17.0%		15.1%		2.2%	
	*n*	%	*n*	%	*n*	%	*n*	%	*n*	%
Head and neck	505	33.8%	403	79.8%	50	9.9%	51	10.1%	1	0.2%
Oral	83	5.5%	58	69.9%	15	18.1%	10	12.0%	0	0.0%
Sinonasal	412	27.5%	342	83.0%	32	7.8%	37	9.0%	1	0.2%
Pharynx/glottis	10	0.7%	3	30.0%	3	30.0%	4	40.0%	0	0.0%
Gastrointestinal tract	76	5.1%	27	35.5%	10	13.2%	39	51.3%	0	0.0%
Anorectal tract	248	16.6%	104	41.9%	70	28.2%	73	29.4%	1	0.4%
Rectum	136	9.1%	45	33.1%	40	29.4%	51	37.5%	0	0.0%
Anus	112	7.5%	59	52.7%	30	26.8%	22	19.6%	1	0.9%
Female genital tract	640	42.8%	433	67.7%	122	19.1%	54	8.4%	31	4.8%
Vulva	458	30.6%	301	65.7%	101	22.1%	26	5.7%	30	6.6%
Vagina	157	10.5%	111	70.7%	20	12.7%	25	15.9%	1	0.6%
Other	25	1.7%	21	84.0%	1	4.0%	3	12.0%	0	0.0%
Urinary tract	16	1.1%	11	68.8%	0	0.0%	5	31.3%	0	0.0%
Respiratory tract	11	0.7%	5	45.5%	2	18.2%	4	36.4%	0	0.0%

**Table 3:** Overall 1-, 2-, and 5-year and median survival and 5-year relative survival for patients with mucosal melanoma by stage and tumour site.

	1-Year OS		2-Year OS		5-Year OS		Median OS		5-Year RS	
	%	95%CI	%	95%CI	%	95%CI	Years	95%CI	%	95%CI
All	67.2	(64.7–69.5)	44.4	(41.9–46.9)	23.8	(21.6–26.0)	1.7	(1.6–1.8)	29.0	(26.2–31.8)
**Tumour stage**										
Local/locally advanced	77.2	(74.5–79.7)	55.2	(52.1–58.3)	30.8	(27.9–33.7)	2.4	(2.1–2.7)	37.5	(33.7–41.2)
Locoregional spread disease	62.2	(55.9–67.8)	33.1	(27.4–38.9)	14.0	(10.0–18.8)	1.3	(1.1–1.6)	17.4	(12.1–23.4)
Distant spread disease	31.4	(25.5–37.5)	12.8	(8.9–17.6)	5.2	(2.8–8.8)	0.6	(0.4–0.7)	6.7	(3.4–11.5)
**Tumour site**										
Head and neck	68.7	(64.5–72.6)	47.7	(43.3–52)	24.7	(20.9–28.6)	1.9	(1.7–2.1)	30.8	(26.0–35.7)
Oral	77.1	(66.5–84.7)	54.1	(42.8–64.1)	28.4	(18.9–38.5)	2.6	(1.7–3.5)	31.0	(20.2–42.3)
Sinonasal	67.0	(62.2–71.3)	46.4	(41.5–51.1)	23.8	(19.7–28.1)	1.8	(1.5–2.1)	30.8	(25.4–36.3)
Gastrointestinal tract	36.8	(26.2–47.5)	19.7	(11.7–29.3)	13.2	(6.7–21.8)	0.7	(0.5–0.9)	17.2	(9.1–27.4)
Anorectal tract	58.5	(52.1–64.3)	30.2	(24.6–36)	14.8	(10.6–19.6)	1.2	(1.0–1.4)	18.3	(12.9–24.4)
Rectum	52.9	(44.2–60.9)	23.5	(16.8–30.9)	11.5	(6.8–17.6)	1.0	(0.8–1.3)	13.9	(7.9–21.5)
Anus	65.2	(55.6–73.2)	38.4	(29.4–47.3)	18.9	(12.2–26.8)	1.6	(1.2–1.8)	23.9	(15.0–34.0)
Female genital tract	73.8	(70.2–77.0)	50.5	(46.5–54.3)	27.8	(24.4–31.4)	2.0	(1.8–2.5)	33.4	(28.8–38.0)
Vulva	79.0	(75.0–82.5)	59.0	(54.3–63.3)	34.5	(30.0–38.9)	2.9	(2.5–3.4)	41.4	(35.5–47.2)
Vagina	58.0	(49.8–65.2)	26.1	(19.5–33.2)	9.5	(5.6–14.7)	1.1	(1.0–1.4)	12.1	(7.0–18.8)
Urinary tract	68.8	(40.5–85.6)	56.3	(29.5–76.2)	31.3	(11.4–53.6)	2.8	(0.5–5.1)	38.0	(12.4–63.9)
Respiratory tract	18.2	(2.9–44.2)	18.2	(2.9–44.2)	9.1	(0.5–33.3)	0.4	(0.2–0.9)	14.1	(1.2–41.6)

**Table 4:** Univariable and multivariable analysis on the impact of the period of diagnosis on the survival of patients with mucosal melanoma.

	Univariable Analyses			Multivariable Analyses: Complete Model			Multivariable Analyses: Definitive Model Stratified for Surgery		
	HR	95%-CI	*p*	HR	95%-CI	*p*	HR	95%-CI	*p*
**Period of diagnosis**									
1990–2013	ref			ref			ref		
2014–2019	0.85	(0.74–0.98)	**0.02**	0.82	(0.71–0.95)	**<0.01**	0.82	(0.71–0.95)	**<0.01**
**Sex**									
Male	1.32	(1.17–1.49)	**<0.01**	1.11	(0.96–1.28)	0.17			
Female	ref			ref					
**Age at diagnosis (years)**									
0–59	ref			ref			ref		
60–69	1.23	(1.03–1.47)	**0.02**	1.27	(1.06–1.52)	**<0.01**	1.33	(1.11–1.59)	**0.01**
70–79	1.54	(1.31–1.82)	**<0.01**	1.51	(1.28–1.78)	**<0.01**	1.59	(1.35–1.87)	**<0.01**
≥80	2.28	(1.94–2.68)	**<0.01**	2.26	(1.91–2.67)	**<0.01**	2.34	(1.98–2.76)	**<0.01**
**Tumour site**									
Head and neck	ref			ref			ref		
Gastrointestinal tract	1.87	(1.45–2.40)	**<0.01**	1.27	(0.97–1.65)	0.08	1.22	(0.95–1.60)	0.12
Anorectal tract	1.39	(1.18–1.63)	**<0.01**	1.15	(0.96–1.38)	0.13	1.12	(0.95–1.33)	0.17
Female genital tract	0.86	(0.76–0.97)	**0.02**	0.89	(0.76–1.05)	0.17	0.82	(0.72–0.93)	**<0.01**
Urinary tract	0.93	(0.53–1.61)	0.79	0.85	(0.49–1.50)	0.58	0.83	(0.47–1.44)	0.51
Respiratory tract	2.79	(1.53–5.08)	**<0.01**	2.42	(1.30–4.50)	**<0.01**	2.45	(1.32–4.54)	**<0.01**
**Tumour stage**									
Local/locally advanced disease	ref			ref			ref		
Locoregional spread disease	1.67	(1.44–1.93)	**<0.01**	1.55	(1.33–1.80)	**<0.01**	1.61	(1.38–1.87)	**<0.01**
Distant spread disease	3.56	(3.05–4.15)	**<0.01**	2.73	(2.27–3.30)	**<0.01**	2.56	(2.13–3.09)	**<0.01**
Unknown	1.98	(1.39–2.82)	**<0.01**	1.75	(1.22–2.51)	**<0.01**	1.68	(1.17–2.42)	**0.01**
**Surgery**									
No	ref			ref					
Yes	0.31	(0.27–0.35)	**<0.01**	0.45	(0.38–0.52)	**<0.01**			
**Hospital of first surgery**									
Academic centre	ref								
General hospital	0.97	(0.84–1.11)	0.63						
Unknown	1.03	(0.86–1.23)	0.77						
**Radiotherapy**									
No	ref			ref					
Yes	1.19	(1.06–1.34)	**<0.01**	1.05	(0.92–1.21)	0.48			
**Chemotherapy**									
No	ref			ref					
Yes	1.94	(1.38–2.73)	**<0.01**	0.82	(0.57–1.18)	0.29			
**Immune and targeted therapy**									
No	ref			ref			ref		
Yes	1.39	(1.02–1.90)	**<0.01**	0.55	(0.38–0.79)	**<0.01**	0.60	(0.42–0.86)	**0.01**
**Hospital of first contact**									
Academic centre	ref			ref					
General hospital	1.08	(0.92–1.27)	0.34	1.08	(0.92–1.27)	0.34			
Unknown	0.42	(0.10–1.69)	0.22	0.42	(0.10–1.69)	0.22			

## Data Availability

The dataset analysed for the current study is not publicly available due to the potentially identifiable nature of the data. However, fully deidentified data can become available from the corresponding author upon reasonable request.

## Supplementary Materials

The following supporting information can be downloaded at: https://www.mdpi.com/article/10.3390/cancers15051541/s1. Table S1. Grouping of mucosal melanomas according to the International Classification of Diseases for Oncology (ICD-O-3) topography coding. Table S2. Primary treatment of mucosal melanoma by stage of disease
