# Supplementary material for: Trends in Incidence and Survival of 1496 Patients with Mucosal Melanoma in The Netherlands (1990–2019)
Source: Cancers (Basel). 2023 Feb 28;15(5):1541. doi: 10.3390/cancers15051541 (PMC10001276; doi:10.3390/cancers15051541)
Supplement: Supplementary file 1 [file cancers-15-01541-s001.zip › cancers-2234567-supplementary.pdf]

Supplementary materials

Table S1. Grouping of mucosal melanoma according to the International Classification of Diseases for Oncology (ICD-O-3) topography coding

ICD-O-3 topograhy code - subsite	Site	Subcategory
C00.3-external lip upper	head and neck	oral
C00.4-external lip lower	head and neck	oral
C01.9-base of tongue, NOS	head and neck	oral
C02.1-border of tongue	head and neck	oral
C03.0-upper gum	head and neck	oral
C03.1-lower gum	head and neck	oral
C03.9-gum, NOS	head and neck	oral
C04.0-anterior floor of mouth	head and neck	oral
C04.1-lateral floor of mouth	head and neck	oral
C05.0-hard palate	head and neck	oral
C05.1-soft palate, NOS	head and neck	oral
C05.8-overlapping lesion of palate	head and neck	oral
C06.0-cheek mucosa	head and neck	oral
C06.1-vestibule of mouth	head and neck	oral
C06.2-retromolar area	head and neck	oral
C06.8-mouth, NOS	head and neck	oral
C09.9-tonsil, NOS	head and neck	oral
C11.0-superior wall of nasopharynx	head and neck	pharynx and glottis
C11.1-posterior wall of nasopharynx	head and neck	pharynx and glottis
C11.3-anterior wall of nasopharynx	head and neck	pharynx and glottis
C11.8-overlapping lesion of nasopharynx	head and neck	pharynx and glottis
C11.9-nasopharynx	head and neck	pharynx and glottis
C12.9-pyriform sinus	head and neck	sinonasal
C14.0-pharynx, NOS	head and neck	pharynx and glottis
C30.0-nasal cavity (excludes nose, NOS C76.0)	head and neck	sinonasal
C30.1-middle ear	head and neck	sinonasal
C31.0-maxillary sinus	head and neck	sinonasal
C31.1-ethmoid sinus	head and neck	sinonasal
C31.2-frontal sinus	head and neck	sinonasal
C31.3-sphenoid sinus	head and neck	sinonasal
C31.8-overlapping lesion of accessory sinuses	head and neck	sinonasal
C31.9-accessory sinus, NOS	head and neck	sinonasal
C32.0-glottis	head and neck	pharynx and glottis
C32.1-supraglottis	head and neck	pharynx and glottis
C15.0-cervical esophagus	gastrointestinal tract	-

C15.3-upper third of esophagus	gastrointestinal tract	-
C15.4-middle third of esophagus	gastrointestinal tract	-
C15.5-lower third of esophagus	gastrointestinal tract	-
C15.9-esophagus NOS	gastrointestinal tract	-
C16.0-stomach, cardia	gastrointestinal tract	-
C16.2-body of stomach	gastrointestinal tract	-
C16.3-gastric antrum	gastrointestinal tract	-
C16.5-lesser curvature of stomach	gastrointestinal tract	-
C16.6-greater curvature of stomach	gastrointestinal tract	-
C16.8-overlapping lesion of stomach	gastrointestinal tract	-
C16.9-stomach NOS	gastrointestinal tract	-
C17.0-duodenum	gastrointestinal tract	-
C17.1-jejunum	gastrointestinal tract	-
C17.2-ileum	gastrointestinal tract	-
C17.9-small intestine, NOS	gastrointestinal tract	-
C18.2-ascending colon; right colon	gastrointestinal tract	-
C18.7-simoid colon	gastrointestinal tract	-
C25.0-head of pancreas	gastrointestinal tract	-
C25.9-pancreas, NOS	gastrointestinal tract	-
C20.9-rectum, NOS	anorectal tract	rectum
C21.0-anus, NOS	anorectal tract	anus
C21.1-anal canal	anorectal tract	anus
C21.2-cloacogenic zone	anorectal tract	anus
C21.8-overlapping lesion of rectum, anus, anal canal	anorectal tract	anus
C51.0-labium majus	female genital tract	vulva
C51.1-labium minus	female genital tract	vulva
C51.2-clitoris	female genital tract	vagina
C51.8-overlapping lesion of vulva	female genital tract	vulva
C51.9-vulva NOS	female genital tract	vulva
C52.9-vagina NOS	female genital tract	vagina
C53.0-endocervix	female genital tract	other
C53.8-overlapping lesion of cervix uteri	female genital tract	other
C53.9-cervix uteri NOS	female genital tract	other
C55.9-uterus NOS	female genital tract	other
C56.9-ovary	female genital tract	other
C57.7-female genital tract, NOS	female genital tract	other
C57.8- overlapping lesion of female genital organs	female genital tract	other
C67.1-dome, bladder	urinary tract	-
C67.4-posterior wall bladder	urinary tract	-
C67.5-neck of bladder	urinary tract	-
C67.8-overlapping lesion of bladder	urinary tract	-
C67.9-bladder, NOS	urinary tract	-
C68.0-urethra	urinary tract	-
C33.9-trachea	respiratory tract	-

C34.1-upper lobe, lung	respiratory tract	-
C34.2-middle lobe, lung	respiratory tract	-
C34.3-lower lobe, lung	respiratory tract	-

Table S2. Primary treatment of mucosal melanoma by stage of disease

	total		local/locally advanced disease		locoregional spread disease		distant spread disease		unknown	
	1496		983		254		226		33	
	n	%	n	%	n	%	n	%	n	%
**Surgery**										
No	344	23.0%	129	13.1%	51	20.1%	151	66.8%	13	39.4%
Yes	1152	77.0%	854	86.9%	203	79.9%	75	33.2%	20	60.6%
**Radiotherapy**										
No	1036	69.3%	657	66.8%	181	71.3%	171	75.7%	27	81.8%
Yes	460	30.7%	326	33.2%	73	28.7%	55	24.3%	6	18.2%
**Systemic therapy**										
No	1409	94.2%	974	99.1%	240	94.5%	162	71.7%	33	100.0%
Yes	87	5.8%	9	0.9%	14	5.5%	64	28.3%	0	0.0%
**Chemotherapy**										
No	1462	97.7%	979	99.6%	250	98.4%	200	88.5%	33	100.0%
Yes	34	2.3%	4	0.4%	4	1.6%	26	11.5%	0	0.0%
**Immune and targeted therapy**										
No	1443	96.5%	978	99.5%	244	96.1%	188	83.2%	33	100.0%
Yes	53	3.5%	5	0.5%	10	3.9%	38	16.8%	0	0.0%
